# Somatosensory-evoked response induces extensive diffusivity and kurtosis changes associated with neural activity in rodents

**DOI:** 10.1162/imag_a_00445

**Published:** 2025-02-18

**Authors:** Andreea Hertanu, Tommaso Pavan, Ileana O. Jelescu

**Affiliations:** Department of Radiology, Lausanne University Hospital (CHUV) and University of Lausanne, Lausanne, Switzerland

**Keywords:** diffusion MRI, functional MRI, microstructure, time-dependence, cell-membrane permeability

## Abstract

Neural tissue microstructure is dynamic during brain activity, presenting changesin cellular morphology and membrane permeability. The sensitivity of diffusionMRI (dMRI) to restrictions and hindrances in the form of cell membranes orsubcellular structures enables the exploration of brain activity under a newparadigm, offering a more direct functional contrast than itsblood-oxygenation-level-dependent (BOLD) counterpart. The current work aims atprobing Mean Diffusivity (MD) and Mean Kurtosis (MK) changes and theirtime-dependence signature across various regions in the rat brain duringsomatosensory processing and integration, upon unilateral forepaw stimulation.We report a*decrease*in MD in the contralateral primarysomatosensory cortex, forelimb region (S1FL), previously ascribed to cellularswelling and increased tortuosity in the extracellular space, paralleled by apositive BOLD response. For the first time, we also report a paired*decrease*in MK during stimulation in S1FL, suggestingincreased membrane permeability. This observation was further supported by thereduction in exchange time estimated from the kurtosis time-dependence analyses.Conversely, the secondary somatosensory cortex and subcortical areas, formerlyreported as responsive to sensory stimulation in rodents (thalamus, striatum,hippocampal subfields), displayed a marked MD and MK*increase,*paralleled by a weak-to-absent BOLD response. Overall, MD and MK uncoveredfunctional-induced changes with higher sensitivity than BOLD. Although the exactorigin of the MD and MK increase is yet to be unraveled, the potential of dMRIto provide complementary functional insights, even below the BOLD detectionthreshold, has been showcased.

## 1 Introduction

Neural activity involves the coordinated interplay of neurovascular andneurometabolic coupling mechanisms, where electrical signals in neurons triggerchanges in blood flow and metabolism (Sokoloff, 1978). This complex process encompasses action potentials,synaptic transmission, and the integration of excitatory and inhibitory signals,contributing to essential brain functions.

By measuring the regional disparity in oxygen consumption and supply, theblood-oxygenation-level-dependent (BOLD) technique harnesses the interaction betweenhemodynamic and metabolic factors and provides a surrogate for brain activity (Ogawa et al., 1990). Since itsinception, functional Magnetic Resonance Imaging (fMRI) based on the BOLD effect(BOLD-fMRI) has become widely used and applied in the field of neuroscience. Overthe years, BOLD-fMRI has been instrumental in investigating various aspects of brainfunction, from the intricacies of memory and attention to the examination ofneurological disorders (Matthews& Jezzard, 2004). Its versatility extends beyond clinical realms,contributing to the exploration of responses to sensory stimuli, decision-making,and social cognition (Morita et al.,2016).

A less well-known coupling linking neural activity and the mechanical tissueproperties was illustrated by several groups reporting transient activity-dependentneuronal and glial swelling (Andrew& Macvicar, 1994;Chéreau et al., 2017;Costa et al., 2018;Fraser & Huang, 2004;Ling et al., 2020;Kwon et al., 2023;Saly & Andrew, 1993;Sun & Wu, 2001;Tasaki & Byrne, 1992). The neuromorphologicalcoupling was hypothesized as an underlying mechanism for another type of fMRIcontrast based on diffusion MRI (dMRI), thought to provide a more direct mapping ofneural activity as compared to BOLD (Asoet al., 2013;Le Bihan etal., 2006). Indeed, the spatial specificity and sensitivity of BOLD-fMRIis a challenging and stimulating debate (Norris, 2012) heightened by nonlinear, time-varying, andpartially unknown crosstalk between hemodynamic, metabolic, and neuronal activity(Buxton et al., 1998;Friston et al., 2000;Sundqvist et al., 2022). On theother hand, diffusion functional MRI (dfMRI) (Abe, Tsurugizawa, et al., 2017;Aso et al., 2013;Darquié et al., 2001;Le Bihan et al., 2006;Tsurugizawa et al., 2013)leverages the sensitivity of the diffusion-weighted signal to microstructuralfeatures and provides a distinctive approach to probe cellular morphologyfluctuations accompanying neural activity.

Owing to methodological hurdles, dfMRI has been subject to controversy over itsability to reflect neuromorphological changes rather than plain vascularmodifications (i.e., BOLD contribution) (Bai et al., 2016;Kuroiwa et al., 2014;Miller et al., 2007;Williams et al., 2016). However, recent studies withoptimal signal-to-noise ratio (SNR) (Nunes et al., 2019), ultra-high temporal resolution (Nunes et al., 2021), and carefulacquisition schemes minimizing vascular confounds (Olszowy et al., 2021;Nguyen-Duc, et al., 2025) have shown that non-vascularinformation can be gleaned from dfMRI. Notably, time-courses of apparent diffusioncoefficient (ADC) estimated from pairs of diffusion-weighted images acquired in aninterleaved fashion, rather than time-courses of diffusion-weighted signals, cancelout most vascular contributions to dfMRI through T_2_-weighting (Darquié et al., 2001;Nguyen-Duc, et al., 2025;Nunes et al., 2021;Olszowy et al., 2021;Yacoub et al., 2008). The typicalreduction in ADC during neuronal activation reported in such dfMRI studies wasattributed to increased tortuosity in the extracellular space and cellular swellingand was detected during brain activity even in the absence of neurovascular coupling(Flint et al., 2009;Lin et al., 2014;Spees et al., 2013;Tirosh & Nevo, 2013;Tsurugizawa et al., 2013).

Furthermore, impairment of the water transport mechanisms taking place through theastrocytic aquaporin channels reflected as a decrease in ADC in the living rodentbrain (Badaut et al., 2011;Pavlin et al., 2017), while theaddition of neural activity inhibitors in rat brain organotypic cortical cultures(Bai et al., 2019) led to aconsiderable decrease in the trans-membrane active water cycling mechanisms measuredthrough relaxometry.

Time-dependent analyses can provide insights into both tissue microstructure andpermeability characteristics (Lee etal., 2020). For instance, at short to intermediate diffusion times, watermolecules may not fully explore their environment, leading to non-Gaussiandiffusion. In the long-time limit, diffusion becomes Gaussian as the water moleculeshave interacted with all of the environment equally (so-called coarse-graining). Theway mean diffusivity (MD) and mean kurtosis (MK) vary with diffusion time (i.e., thefunctional form of this time-dependence) informs on the type of structural disorderthe molecules are encountering. For example, the functional form associated with1D-disorder (Novikov et al.,2014) applies to intra-cellular water in axons, dendrites, or other cellprocesses and can provide information on beading or spines. The functional formassociated with 2D-3D structural disorder (Novikov et al., 2014) applies to extra-cellular water andcan provide information on outer axon diameter or other characteristic lengths ofthe extracellular space. For a given family of structural disorder, the samefunctional form will govern both MD and MK time-dependence. Additionally, MKtime-dependence may stem from inter-compartmental water exchange, which can beestimated using the Kärger model (Kärger, 1985).

In this context, the goal of our work was to investigate how brain activity changesMD and MK values (at each diffusion time) but potentially also changes theirdiffusion time-dependence signature, which could provide additional insight intoactivity-driven microstructural changes. The fast kurtosis method (Hansen, Lund, et al., 2016;Hansen, Shemesh, et al., 2016)facilitated the estimation of MD and MK for multiple diffusion times from a reducednumber of diffusion-weighted images, compatible with the brief rest and stimulusintervals typical of task fMRI block designs. The rat forepaw unilateral electricalstimulation was chosen due to its widespread adoption and extensive use as anexperimental model for the exploration of brain function along the somatosensorypathways in rodents. We aimed at probing brain-wide MD and MK dynamics, includingvarious cortical and subcortical structures reported in the literature as part ofsomatosensory processing and integration.

## 2 Materials and Methods

### 2.1 Animal preparation and anesthesia

Experiments were approved by the local Service for Veterinary Affairs of thecanton of Vaud. Ten female Wistar rats (Charles River, France) (236 ± 14g) were scanned on a 14.1T MRI system (Bruker BioSpin), using a home-builtsurface quadrature transceiver RF-coil. The anesthesia, stimulation, and animalpreparation protocols were similar to those outlined in a previous study (Reynaud et al., 2019).Isoflurane anesthesia (4% for induction and 2% during setup) was used whilepositioning the rat into a custom-made MRI cradle. Electrical stimulation wasenabled using two pairs of stainless-steel electrodes inserted in the ratforepaws, between digits 2 and 3, and digits 4 and 5, respectively. A catheterwas inserted subcutaneously on the back of the animal for medetomidineadministration. Once the setup was finalized, an initial bolus of 0.1 mg/kg ofmedetomidine was injected, while gradually reducing the isoflurane anesthesia.Ten minutes later, the isoflurane was completely interrupted, and another 5 minlater a continuous medetomidine infusion at 0.1 mg/kg/h was started. The totalsedation time under medetomidine was around two hours and a half, with anaverage respiration rate of 67 ± 18 breaths per minute and an averagetemperature of 38 ± 0.5 °C across all rats and acquisitions. Anoxygen/air supply of 20%/80% was maintained throughout the experiment. At theend of the experiment, animals were woken up by an intramuscular injection ofatipamezole at 0.5 mg/kg.

### 2.2 Paradigm design

A custom-triggering routine making use of the Transistor-Transistor Logic (TTL)pulse output delivered by the MRI scanner was implemented to generate a blockparadigm for the dMRI and BOLD-fMRI acquisitions with alternating cycles of 28 sof rest and 28 s of stimulation. The stimulation consisted of 0.3 ms pulses at 2mA and 8 Hz frequency and was delivered by an A365 stimulus isolator interfacedwith a DS8000 digital stimulator (World Precision Instruments, Stevenage, UK).Twenty-eight images for each rest and stimulus intervals were acquired duringthe BOLD-fMRI (TR = 1 s), and 14 during the dMRI (TR = 2 s) blockdesigns, respectively.

### 2.3 Data acquisition

**Fig. 1.**
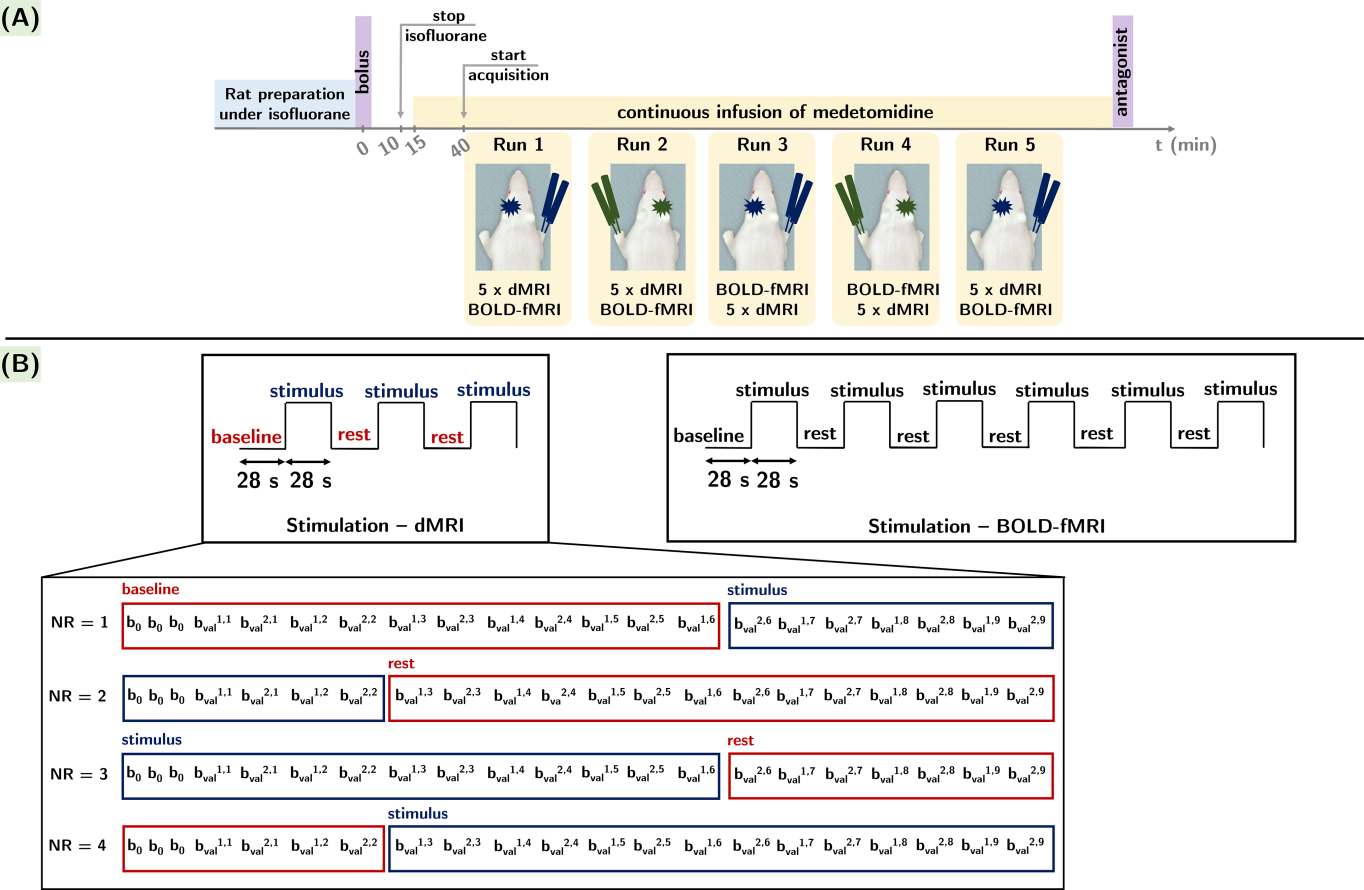
Data acquisition protocol. (A) Experimental timeline. (B) Each functionalrun included five different diffusion sequences with varying diffusiontime values and one BOLD-fMRI sequence. For each diffusion time, thetiming of the three epochs of alternating 28 s rest and 28 s stimulusintervals was set such as to acquire NR = 2 repetitions of 21measurements*—*nine directions for twob-values (b_val_^i,j^with i = 1; 2ms/µm^2^and j = 9 directions) and threeb_0_acquisitions for each condition (rest vs.stimulation). One BOLD-fMRI acquisition included the acquisition of 336volumes equally spread across six epochs of alternating 28 s rest and 28s stimulus intervals.

Figure 1details theexperimental design. The acquisition of functional runs comprising both dMRI andBOLD-fMRI sequences started 30 min after isoflurane cessation (Fig. 1A). During that 30-minwait time for isoflurane clearance, a B_0_map for field-map basedshimming of the rat brain with default acquisition parameters and aT_2_-weighted (T_2_w) structural acquisition foranatomical reference were performed. The T_2_w structural scan wasacquired using a 2D multi-slice RARE sequence, with the following parameters: TE= 6 ms, TR = 2.5 s, matrix size 160 × 160 × 45,in-plane resolution 125 × 125 μm^2^, slice thickness 0.5mm, and RARE factor = 1.

Five functional runs were acquired per rat (only four runs for two rats). Everyfunctional run was composed of five dMRI acquisitions with different diffusiontimes (Δ = 9.5; 15; 20; 25; 30 ms) and one BOLD-fMRI acquisition.From one functional run to another, the forepaw used for stimulation wasalternated and within each run, the order of the dMRI/BOLD-fMRI acquisitions wasrandomized. When tallying the total number of animals and functional runs, 24dMRI datasets per diffusion time and per stimulated forepaw and 24 BOLD-fMRItime-series per stimulated forepaw were acquired.Table 1summarizes the acquisition times forthe various sequences in the protocol, as well as the time needed for theacquisition of one functional run and for the entire experimental protocol forone rat.

**Table 1.** Acquisition times for the individual dMRI and BOLD-fMRI sequences, onefunctional run, the B0 map, the T_2_w structural sequence andfinally, for the entire protocol for one rat.

Phase encoding	Forward	Reverse
dMRI acquisition	2 min 48 s	8 s
BOLD-fMRI acquisition	5 min 36 s	10 s
1 run (5 × dMRI + fMRI)	19 min 54 s	
B _0_ map	1 min 20 s	
T _2_ w	6 min 40 s	
1 rat dataset	1 h 47 min 30 s	

For each functional run, one dMRI and one BOLD-fMRI reverse-phaseencode acquisition was performed for spatial distortion correction.The total duration of one functional run corresponds to theacquisition times of five dMRI sequences with different diffusiontimes, one BOLD-fMRI sequence, and the two reverse-phase encodeacquisitions. The duration of the entire protocol for one ratincludes the acquisition of the B0 map, the structuralT_2_w image, and five functional runs.

#### 2.3.1 Diffusion MRI

For each diffusion time, a monopolar Pulsed Gradient Spin Echo sequence withEcho-Planar Imaging read-out (PGSE-EPI) was used to acquire two repetitionsof 21 measurements—nine directions for two b-values(b_val_^i,j^with i = 1; 2ms/µm^2^and j = 9 directions) and three b= 0 (b_0_) acquisitions—during each condition (restvs. stimulus). In total, 84 images were equally distributed between threeepochs of alternating rest and stimulus intervals (Fig. 1B) for a total scan time of 2 min 48s. The remaining experimental parameters included: gradient pulse durationδ = 4 ms, TE = 48 ms, TR = 2 s, matrix size 80× 80, 21 slices, in-plane resolution 0.25 × 0.25µm^2^, slice thickness 0.75 µm, and partialFourier acceleration factor of 1.1 in the phase direction. In a separatescan, three b_0_images were acquired with reversed EPIphase-encode direction, without stimulation, for spatial distortioncorrection.

#### 2.3.2 BOLD-fMRI

Data were acquired using a gradient-echo EPI (GE-EPI) sequence with thefollowing parameters: TE = 11.1 ms, TR = 1 s, and matrix size,number of slices, in-plane resolution, and slice thickness matching to thedMRI acquisitions (80 × 80, 21, 250 × 250µm^2^and 750 µm, respectively). A partialFourier acceleration factor of 1.2 was employed for the phase direction. Atotal number of 336 images were acquired during six epochs of alternatingrest and stimulus intervals (Fig.1B), resulting in 168 images for each rest and stimuluscondition, collected in 5 min 36 s. In a separate scan, ten GE-EPI imageswere acquired with reversed EPI phase-encode direction, without stimulation,for spatial distortion correction.

### 2.4 Data processing

#### 2.4.1 Diffusion MRI

**Fig. 2.**
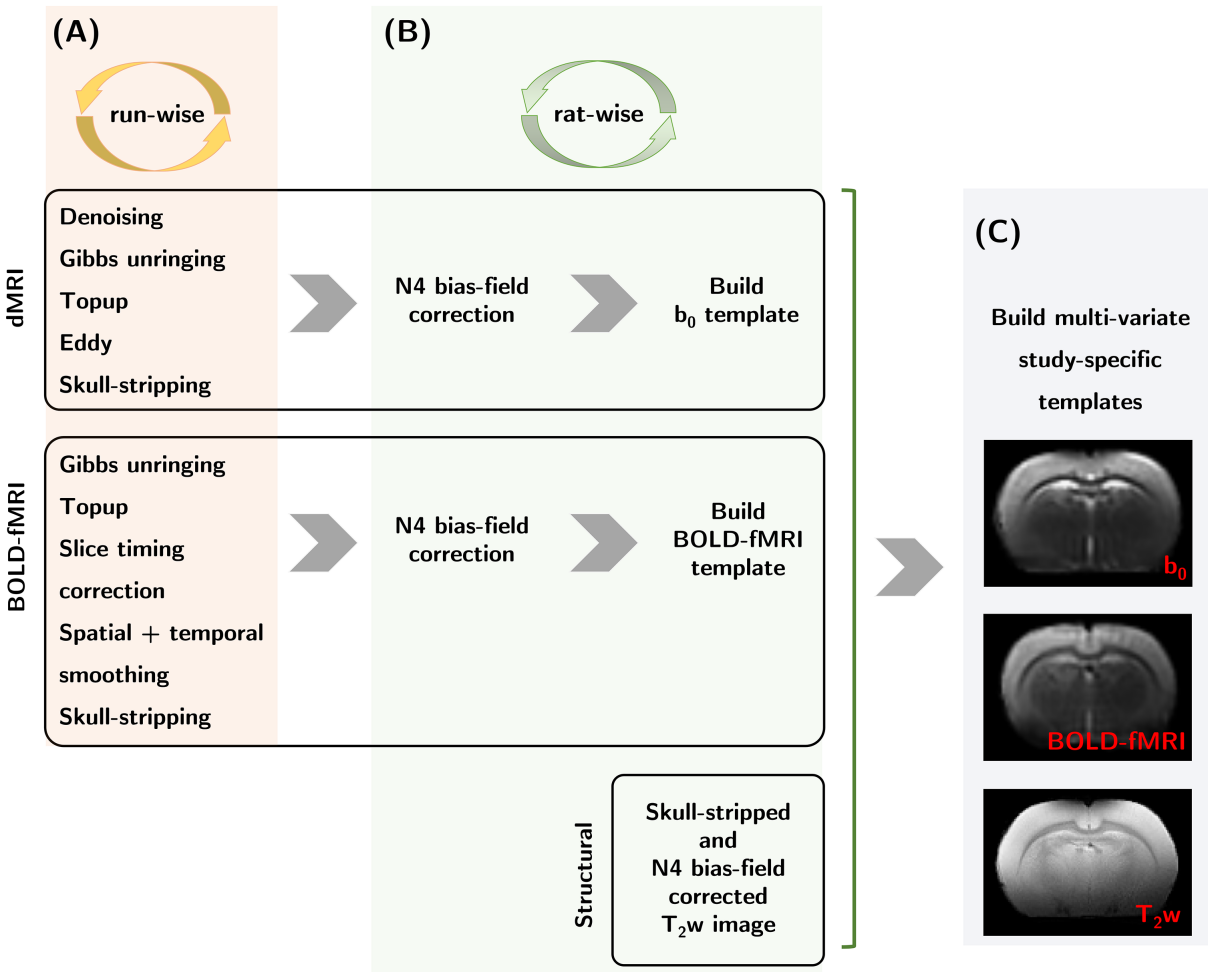
Pre-processing steps. (A) The five diffusion datasets from eachfunctional run were pooled and underwent denoising, Gibbs-unringing,topup and eddy correction, and skull-stripping, while for theBOLD-fMRI time-series topup, slice-timing correction, spatial andtemporal smoothing, and skull-stripping were applied for eachfunctional run. (B) Images were then N4 bias-field corrected and theextracted b_0_images and BOLD-fMRI time-series were usedto build corresponding templates for each rat. (C) Theskull-stripped and N4 bias-field corrected T_2_w imageswere jointly used along with the b_0_and BOLD-fMRIrat-specific templates to construct multi-variate study-specifictemplates intended to facilitate the propagation of brainsegmentations from the WHS T_2_w atlas into the dMRI andBOLD-fMRI individual rat spaces.

Pre-processing steps, conducted in python (v.3.9, Python SoftwareFoundation), are illustrated inFigure 2. The first step (Fig. 2A) included denoising (Tournier et al., 2019;Veraart et al.,2016), Gibbs unringing (Kellner et al., 2016;Tournier et al., 2019),topup (Andersson et al.,2003), and eddy (Andersson & Sotiropoulos, 2016) correction for each runseparately. A rat brain mask was estimated using the atlasbrex (Lohmeier et al., 2019)routine. The two repetitions of diffusion-weighted volumes and theb_0_images acquired for each rest and stimulus condition wereaveraged, and the resulting 19 images per condition were used to perform avoxel-wise estimation (Hansen,Lund, et al., 2016;Hansen, Shemesh, et al., 2016) of MD^rest^,MD^stimulus^, MK^rest^, and MK^stimulus^mapsfor each diffusion time using Matlab (v.R2021b, MathWorks Inc.) scripts(https://github.com/sunenj/Fast-diffusion-kurtosis-imaging-DKI).Voxels yielding unphysical values due to noise or partial volume (e.g.,negative kurtosis or diffusivity) were excluded from further analysis.

#### 2.4.2 BOLD-fMRI

Every BOLD-fMRI time-series went through Gibbs unringing (Kellner et al., 2016;Tournier et al.,2019) and topup correction (Andersson et al., 2003) (Fig. 2A). Prior to the generalized linearmodel (GLM) analysis in SPM12 (http://www.fil.ion.ucl.ac.uk/spm/software/spm12/), time-serieswere slice-timing corrected and minimally smoothed (gaussian smoothingkernel with 0.1 × 0.1 × 1 mm^3^size). High-passfiltering with a cut-off frequency of 0.01 Hz applied on the time-coursesensured the removal of low-frequency noise and drifts. A rat brain mask wasestimated using the atlasbrex (Lohmeier et al., 2019) routine for the BOLD-fMRI volumes. Forthe GLM analysis, a boxcar (Reynaud et al., 2019) response function was used, andstatistically significant voxels were detected using a p-value threshold of0.05 with family-wise error correction. To ensure maximum detectionsensitivity, no threshold was applied on the size of the clustered voxels.Prior to ROI-wise quantifications, a brain mask excluding voxels with asignal lower than 10% of the signal measured in the cortex was generated.This step aimed to remove voxels from deeper brain regions where coilsensitivity was heavily attenuated.

### 2.5 Quantification

ANTs (Avants et al., 2009) wasemployed to generate rat-specific b_0_and BOLD-fMRI templates fromindividual images after applying N4 bias-field correction (Tustison et al., 2010) (Fig. 2B). IndividualT_2_w images were also N4 bias-field corrected and skull-strippedand then used jointly along with the b_0_and BOLD-fMRI rat-specifictemplates to generate study-specific multivariateb_0_/BOLD-fMRI/T_2_w templates (Fig. 2C). The WHS atlas (Papp et al., 2014) wasregistered onto our study-specific T_2_w template, and thetransformation was applied to the WHS atlas segmentation. The multi-variatetemplates facilitated the propagation of the brain segmentations into the nativedMRI and BOLD-fMRI spaces of each rat.

The segmented regions-of-interest (ROIs) are illustrated inFigure S1and included: primary somatosensory cortex, forelimb area (S1FL), secondarysomatosensory cortex (S2), primary motor cortex (M1) as cortical ROIs expectedto be involved in the somatosensory processing, thalamus (Tha), striatum (CPu)and hippocampal subfields (Hip) as subcortical ROIs expected to be part ofsubcortical somatosensory relays, and secondary motor cortex (M2), cingulatecortex (ACC), retrosplenial cortex (RSC), and posterior parietal cortex (PPC) ascontrol cortical ROIs.

Quantifications in each ROI were done in the left hemisphere and were reported ascontralateral when the right forepaw was stimulated (expected response in theleft S1FL), and ipsilateral when the left forepaw was stimulated (little to noresponse expected in left S1FL, as the main response should be in the rightS1FL).

#### 2.5.1 Diffusion MRI

The mean and standard deviation for MD and MK in each ROI were calculated forall diffusion times, runs, and rats in the rest and stimulus conditions andwere then grouped by stimulated forepaw. The average MD and MK values(± standard error) for each forepaw and each condition were computedto yield MD^rest^(∆), MD^stimulus^(∆),MK^rest^(∆), and MK^stimulus^(∆) curvesin all the investigated brain regions contralaterally and ipsilaterally.MD(∆) and MK(∆) time-dependence analyses were conducted inS1FL only. Power-laws for structural disorder (Novikov et al., 2014) and the analyticalformulation of time-dependent kurtosis from the two-compartmentKärger model with exchange (Kärger, 1985) were fit to the experimentalcurves to evaluate potential differences in morphology and permeabilitybetween the rest and stimulus conditions. The following equations were usedfor 1D structural disorder (equations 1and3), 2D-3Dstructural disorder (equations 2and4), and the Kärger exchange model(equation5):

$$\text{MD}(\Delta )≅{\text{D}}_{\infty }+2\text{A}\;\text{*}\;\Delta^{-1/2}$$ (1)

$$\text{MD}(\Delta )≅{\text{D}}_{\infty }+\text{A}\frac{\text{ln}\left(\Delta /{\text{t}}_{\text{c}}\right)}{\Delta }$$ (2)

$$\text{MK}(\Delta )≅{\text{K}}_{\infty }+2\text{A}\;\text{*}\;\Delta^{-1/2}$$ (3)

$$\text{MK}(\Delta )≅{\text{K}}_{\infty }+\text{A}\frac{\text{ln}\left(\Delta /{\text{t}}_{\text{c}}\right)}{\Delta }$$ (4)

$$\text{MK}(\Delta )≅{\text{K}}_{0}\frac{2{\text{t}}_{\text{ex}}}{\Delta }\left[1-\frac{{\text{t}}_{\text{ex}}}{\Delta }\left(1-{\text{e}}^{-\Delta /{\text{t}}_{\text{ex}}}\right)\right]$$ (5)

#### 2.5.2 BOLD-fMRI

Beyond the GLM analysis, the average and standard deviation of the BOLD-fMRIsignal was calculated in each ROI and each volume of the time-series. Foreach time-series, the BOLD signal in each epoch was normalized by theaverage signal in the 14 s preceding the stimulation. Then, the BOLD signalresponse function was calculated by averaging across all epochs, runs, andrats. In a separate analysis, and to make a fairer comparison to the MD andMK measures which result from diffusion-weighted images acquired acrossmultiple blocks of rest and stimulation (i.e., yielding one MD and MKestimate per condition), the BOLD signal was also averaged across alltimepoints of the rest and stimulus intervals, respectively. As a result,one BOLD amplitude estimate (± standard error) per condition wasobtained.

### 2.6 Statistical analyses

All analyses were carried out in RStudio (v.2023.12.1, R Foundation forStatistical Computing), using the lme4 package (Bates et al., 2015). Correction for multiplecomparisons using the false discovery rate (FDR) method was applied on eachmetric (BOLD, MD, and MK) separately.

#### 2.6.1 Diffusion MRI

The mean MD and MK values quantified in each ROI and for each diffusion time,run, rat, and the two conditions were used for the statistical analyses (240total measurements per ROI). The hierarchical structure of our dMRI dataindicated that a mixed-effects model would be the most suitable approach.The choice criteria for the statistical model are detailed inTableS1. Our factorial design included two main effects: the conditionof the rat during the measurement (i.e., rest vs. stimulus) and thediffusion time (five different values) in order to detect statisticallysignificant changes between the rest and stimulus measurements on the onehand, and between measurements at various diffusion times (time-dependence)on the other hand. Two random effects were also considered—the runand the subject indexes. The run index may contribute to random variabilityin the data due to its temporal spacing in relation to the switch-off ofisoflurane anesthesia, slight variations in the temperature, and respirationrate across the runs and biological variability in the rat response to theforepaw stimulus. The subject index represents the random variabilityintroduced by biological differences between the rats.

#### 2.6.2 BOLD-fMRI

The average BOLD amplitudes estimated for the rest and stimulus conditionswere used to determine statistically significant differences in each ROI (48total measurements per ROI). The statistical design including the state ofthe rat during the measurement (i.e., rest vs. stimulus) was the mostsuitable for the evaluation of significant differences as indicated inTableS2.

## 3 Results

### 3.1 BOLD-fMRI time-series analyses

A few examples of spatial localization for the statistically significantactivation clusters resulting from the GLM analysis conducted on the BOLD-fMRItime-series data corresponding to unilateral right forepaw stimulation areillustrated inFigure S2. Globally, the activated area extended over a minimum offive adjacent slices and was mainly localized in the left primary somatosensorycortex. The total number of voxels in the activated region for the 24 datasets(from ten rats) ranged from 14 to 431, with a mean of 179 voxels/cluster. Onaverage, around 80% of the voxels were situated within the primary somatosensorycortex, while the remaining 20% were distributed between the motor cortex (17%)and the secondary somatosensory cortex (3%). Only for five datasets out of 24,the activated area presented sparse voxel distributions in the thalamus(< 6%), and less than five datasets presented sparse voxel distributionsin the cingulate cortex (<3%), retrosplenial cortex (<3%),auditory cortex (<1.1%), hippocampal subfields (<0.5%), andsubicular complex (<0.5%). Percentages are defined with respect to thenumber of significant voxels detected in the whole brain. The GLM analysis didnot unveil any activation clusters in the ipsilateral hemisphere.

### 3.2 Rest vs. stimulus BOLD, MD and MK changes

An example of BOLD image, MD and MK parametric maps for one rat are displayed inFigure 3to illustrate theoverall quality of our data.

**Fig. 3.**
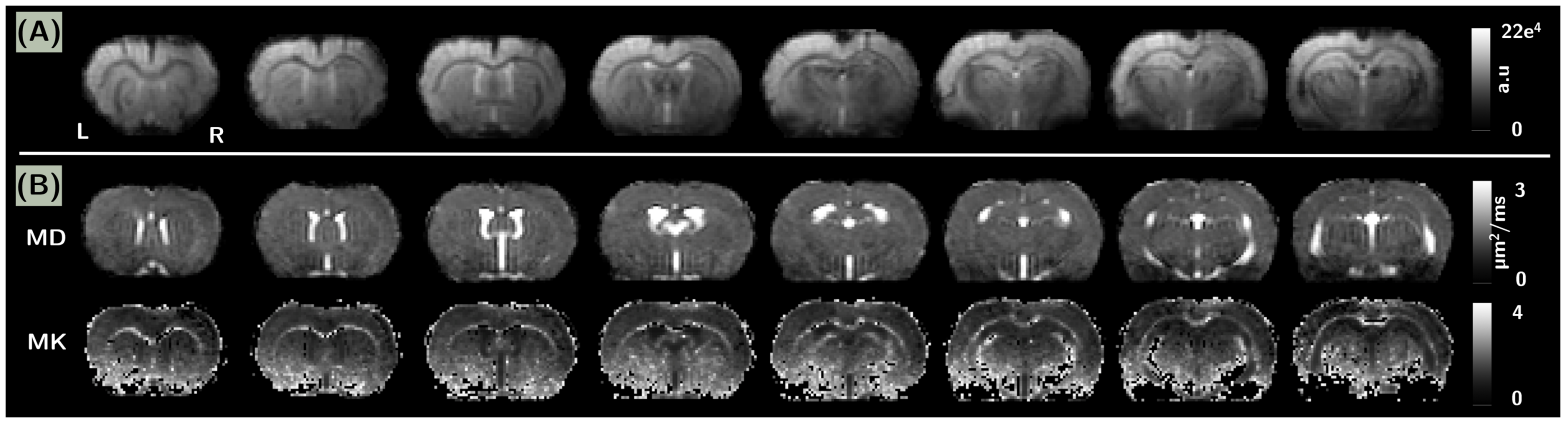
Illustration of the quality of (A) the BOLD-fMRI images after thepre-processing steps and (B) the parametric MD and MK maps, inrostro-caudal slices for one rat. The displayed MD and MK mapscorrespond to a diffusion time of Δ = 9.5 ms.

InTable 2statisticallysignificant amplitude changes in the average BOLD signal, the MD and MK metricsare reported as a percentage of increase or decrease during stimulation withrespect to the rest condition. The associated p-values are reported inTable S3.We underline that the significant BOLD changes result from the analysis of thenormalized time-series signal averaged across each ROI and all rest vs. stimulustimepoints. This approach mirrors the sensitivity of MD and MK estimates, whichare also averaged across entire anatomical ROIs and result fromdiffusion-weighted measurements spread across all the rest vs. stimulustimepoints during each functional acquisition. For MD and MK, the amplitudechange was calculated for each diffusion time and only the absolute maximumvalue was reported. The diffusion times corresponding to the absolute maximumamplitude change are reported inTable S4.

**Table 2.**
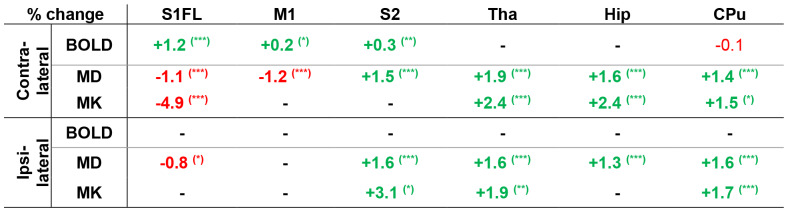
Percentage of signal change during stimulation with respect to theresting condition in the ROIs presenting significant modifications.

The BOLD signal changes represent the average relative differencebetween all rest and stimulus time-points, while the MD and MKchanges represent the maximum relative difference between the restand stimulus conditions obtained across the five diffusion times.The polarity of the signal change is highlighted in colors (greenand red for positive and negative changes, respectively). *p< 0.05, **p < 0.01,***p < 0.001, with FDR correction formultiple comparisons. The CPu presented a weakly significant BOLDsignal decrease (p < 0.05) contralaterally that did notsurvive correction for multiple comparisons. Globally, the absoluteamplitude of the significant MD and MK changes is higher than forBOLD.

#### 3.2.1 Somatosensory cortical brain regions

**Fig. 4.**
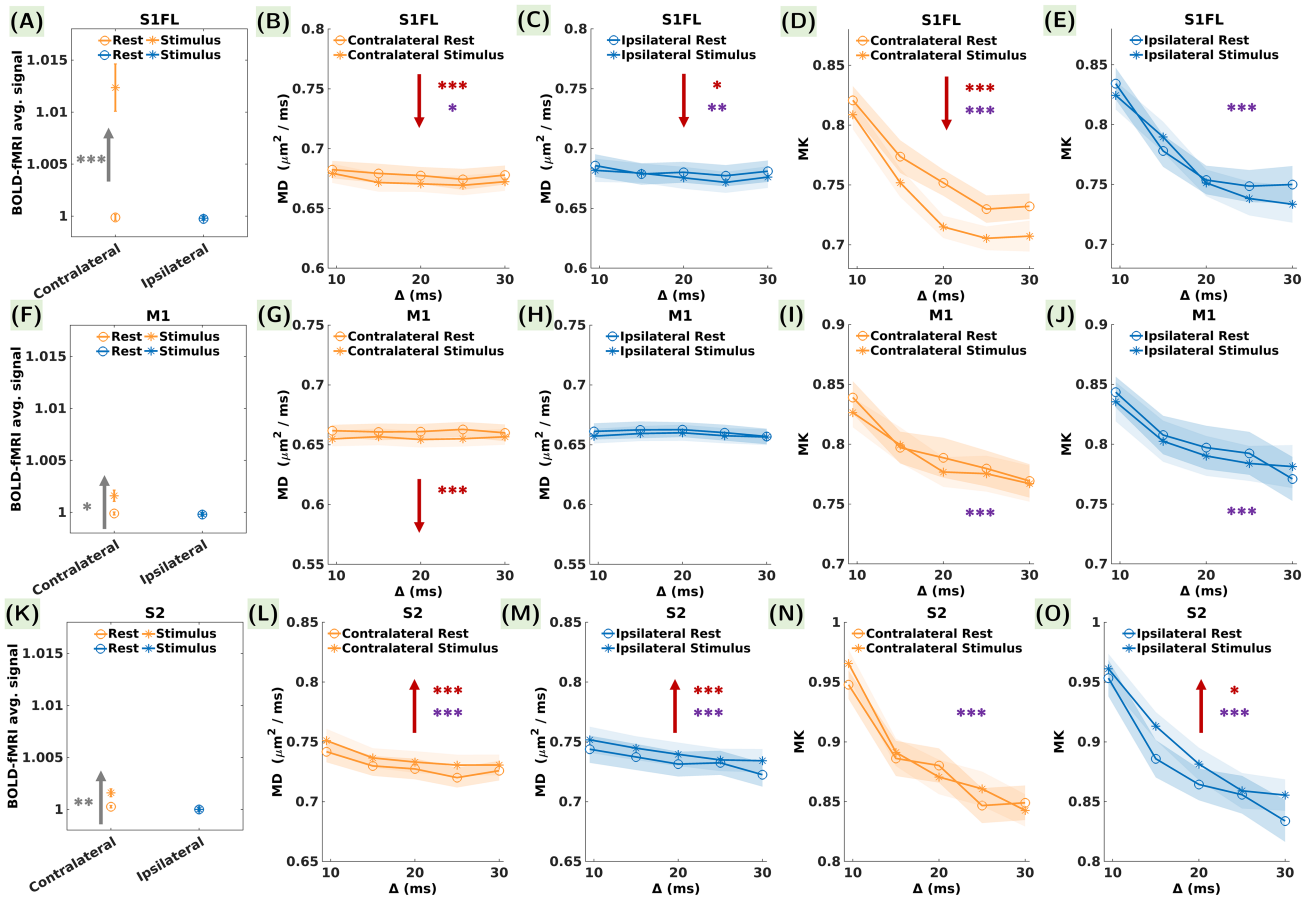
Average BOLD signal, MD, and MK during the rest and stimulusconditions in the cortical somatosensory brain regions: (A-E) S1FL,(F-J) M1, and (K-O) S2, contralaterally (orange) and ipsilaterally(blue). For BOLD, statistical differences between the mean acrossthe rest and stimulus time-points are indicated in gray asterisks(the error bars represent the standard error calculated over 24measurements in each condition). Red and purple asterisks in the MDand MK plots indicate statistically significant differences betweenthe rest and stimulus conditions and between the diffusion timevalues, respectively (the error bars represent the standard errorcalculated over 24 measurements in each condition and each diffusiontime). p-values are reported as: *p < 0.05,**p < 0.01, ***p <0.001 (FDR correction for multiple comparisons). Upwards/downwardsred and gray arrows indicate an increase/decrease in the quantifiedmetrics. The significant average BOLD increase in contralateral S1FLwas accompanied by a significant decrease in MD and MK, while incontralateral M1 a significant average BOLD increase was paired witha significant decrease in MD only. Ipsilaterally, only MD in S1FLdecreased significantly. In contralateral S2 a significant BOLDsignal increase was accompanied by a significant increase in MDcontrasting the results in the primary sensorimotor cortices.Ipsilaterally, a significant increase was found for both MD andMK.

The main brain region involved in processing forelimbstimulation—S1FL—presented an average BOLD signal increase of+1.2% contralaterally (Table 2,Fig.4A). This finding is consistent with the location of thesignificant signal variations detected at the voxel level in the GLManalysis (Fig. S2) and the positive response function calculated in theROI by averaging across all epochs and all rats (Fig.S3A). Ipsilaterally, BOLD signal changes in the whole ROI werenot significant, consistent with the response function displayed inFigureS3A. On the other hand, diffusion metrics presented a significantdecrease in contralateral S1FL during the stimulus condition (Fig. 4B, D), with maximumamplitudes of -1.1% and -4.9% for MD and MK, respectively (Table 2). Ipsilaterally,a weak significant decrease in MD during the stimulation was also found,while no significant changes in MK were detected (Fig. 4C, E).

In contralateral M1, the average BOLD signal increase (Fig. 4F) was significant, but lower inamplitude than in S1FL, reaching only +0.2% (Table 2). The response function inFigureS3Bshows a clear increase in the BOLD signal during thestimulation time-window. Furthermore, a significant MD decrease with anamplitude of -1.2%, remarkably similar in effect size to the MD decrease inS1FL, was measured (Fig.4G,Table2). No significant changes were found ipsilaterally (Fig. 4H, J).

Contralateral S2 also presented an increase in the average BOLD signal duringstimulation, with an amplitude of +0.3% (Fig. 4K,Table 2) and a distinctive positiveresponse function (Fig. S3C). Ipsilaterally, there were nosignificant BOLD signal variations (Table 2). However, a significant increasewas found in contralateral S2 for MD (Fig. 4L), contrasting the trends in S1FLand M1. Remarkably, the MD increase was significant both contra- andipsilaterally (Fig. 4M),with maximum amplitudes of +1.5% and +1.6%, respectively(Table 2). Asignificant MK increase of +3.1% was measured only ipsilaterally(Fig. 4O).

#### 3.2.2 Subcortical somatosensory relays

**Fig. 5.**
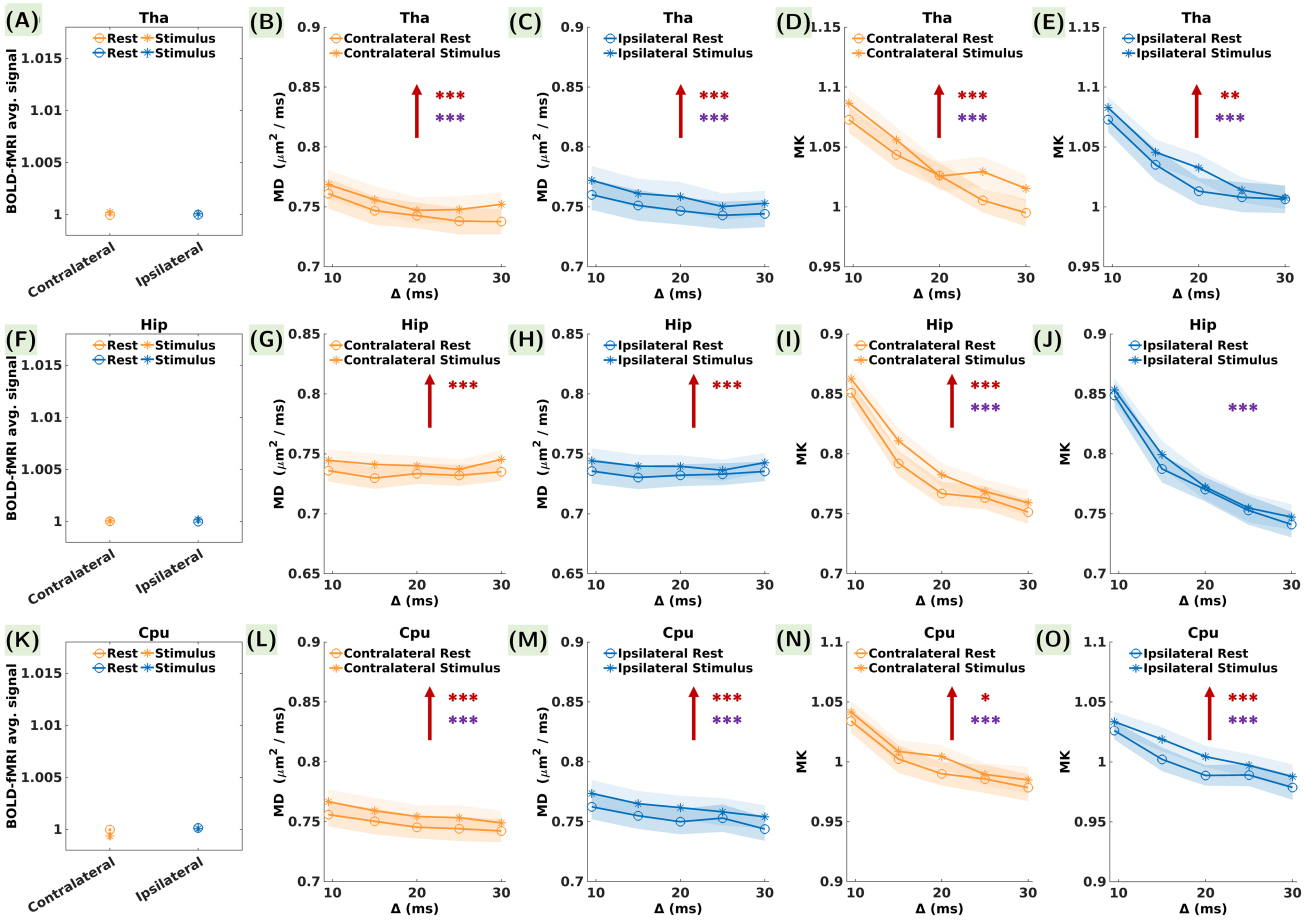
Average BOLD signal, MD, and MK during the rest and stimulusconditions in the subcortical somatosensory relays (A-E) Tha, (F-J)Hip, and (K-O) CPu, contralaterally (orange) and ipsilaterally(blue). No statistical differences between the mean across the restand stimulus time-points were found for BOLD (the error barsrepresent the standard error calculated over 24 measurements in eachcondition). Red and purple asterisks in the MD and MK plots indicatestatistically significant differences between the rest and stimulusconditions and between the diffusion time values, respectively (theerror bars represent the standard error calculated over 24measurements in each condition and each diffusion time). p-valuesare reported as: *p < 0.05, **p <0.01, ***p < 0.001 (FDR correction formultiple comparisons). Upward red arrows indicate an increase in thequantified metrics. Overall, all subcortical ROIs displayed asignificant increase in MD and MK during stimulation bilaterally,except for MK in the ipsilateral Hip and only contralateral CPudisplayed a very weak decrease in BOLD, however non-significantafter FDR correction for multiple comparisons.

In the Tha and the Hip, no significant changes in the average BOLD signalwere found contralaterally and ipsilaterally (Fig. 5A, F) and the response functions didnot display any changes during stimulation (Fig. S3E,F). Remarkably, contralateral CPu displayed a weak decrease inthe average BOLD signal (Fig.5K,Table 2)with an amplitude of -0.1% and a negative response function (Fig.S3G). However, the p-value was no longer significant aftercorrection for multiple comparisons.

Similarly to S2, the subcortical brain regions presented a significantincrease in MD both contralaterally and ipsilaterally (Fig. 5B, C,G, H,L and M), with amplitudes in the range of+1.3 to +1.9%. MK also increased significantly bothcontralaterally and ipsilaterally in all subcortical ROIs (except foripsilateral Hip) with amplitudes ranging from +1.5% to +2.4%(Fig. 5D, E,I, J,N, and O).

#### 3.2.3 Control cortical brain regions

M2, ACC, RSC, and PPC did not display any significant changes in the averageBOLD signal, MD, or MK between the rest and stimulus conditions (Fig.S4).

To summarize, in the primary sensorimotor cortices, we report a significantdecrease in MD in bilateral S1FL and contralateral M1, and a significantdecrease in MK in contralateral S1FL. These changes were paralleled by asignificant BOLD signal increase in contralateral S1FL and M1. In thesecondary somatosensory cortex and subcortical regions, we report anincrease in MD in bilateral S2, Tha, Hip, and CPu, and an increase in MK inbilateral Tha and CPu, ipsilateral S2, and contralateral Hip, while at theBOLD signal level we only found an increase in contralateral S2 and a verymild decrease in contralateral CPu. Globally, significant MD and MK changeswere consistently on the order of 1% or higher throughout the investigatedbrain regions, while the reported significant BOLD signal changes werehigher than 1% in S1FL and lower than 0.5% in S2 and M1.

### 3.3 MD and MK time-dependence

Measurable time-dependence was found for MD and MK bilaterally in most brainregions, except for MD in bilateral M1, M2, and Hip. The corresponding p-valuesare reported inTable S5.

#### 3.3.1 S1FL time-dependence analysis

**Fig. 6.**
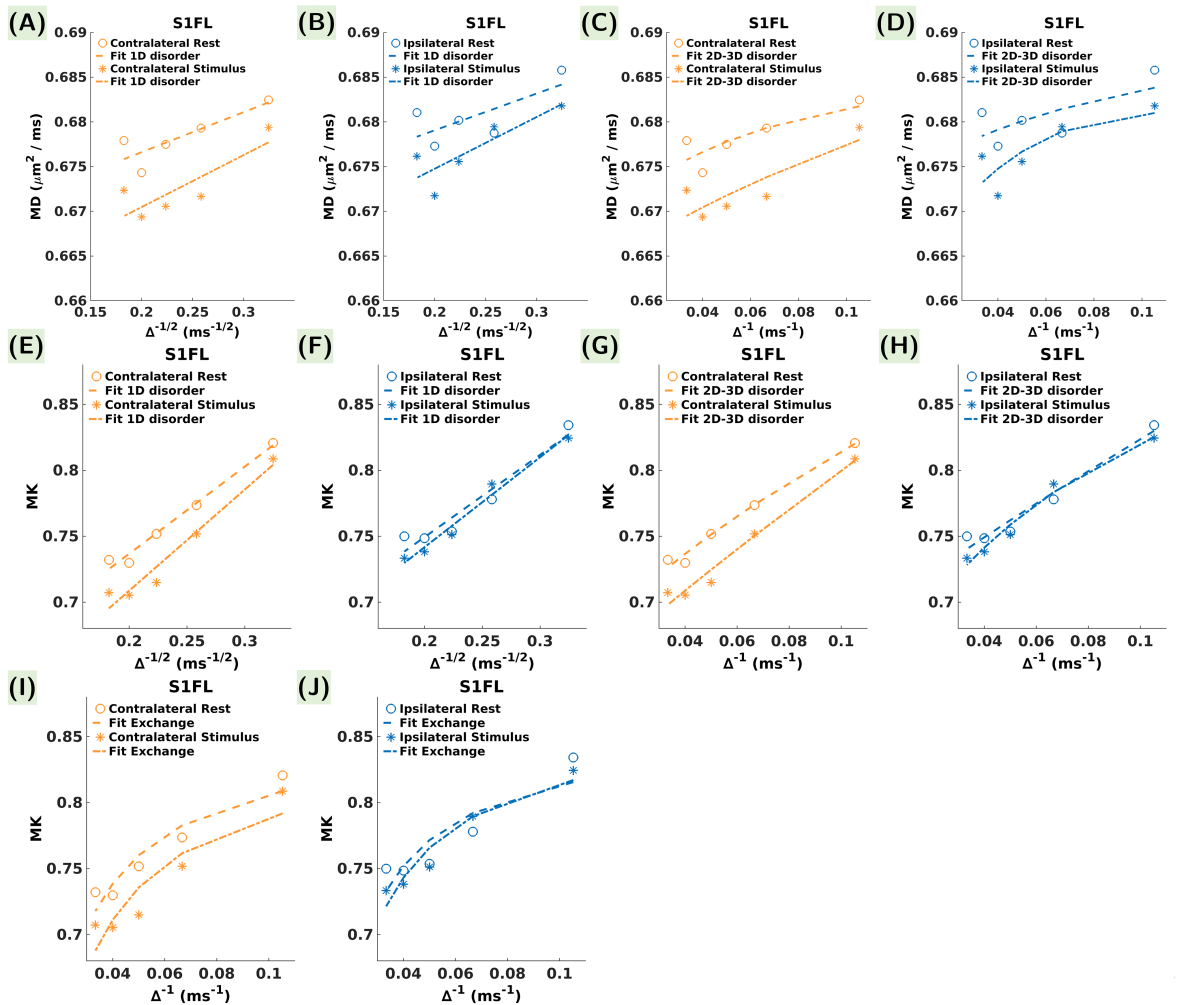
Power-law fit curves (dotted lines) in S1FL for (A, B) structural 1Ddisorder to MD time-dependence, (C, D) structural 2D-3D disorder toMD time-dependence, (E, F) structural 1D disorder to MKtime-dependence, (G, H) structural 2D-3D disorder to MKtime-dependence, and (I, J) the Kärger model kurtosis to MKtime-dependence. The contralateral S1FL is shown in orange, and theipsilateral S1FL in blue.

Analysis of the corrected Akaike information criterion (AICc) values (Table 3), associated withfitting structural disorder models (equations 1and2) to theMD(∆) decay in S1FL at rest and during stimulation (Fig. 6A–D), revealeda superior quality of fit for the 1D structural disorder model. Indeed, wereport lower AICc values along with greater precision in the estimatedcoefficients. Consequently, the underlying mechanisms behind MD(∆)time-dependence align more closely with a description based on 1D structuralirregularities encountered by water molecules within cell processes, ratherthan one based on the 2D-3D geometrical details of the extra-cellular space.Consistency in the fitted A and D_∞_coefficients acrossconditions (contralateral/ipsilateral, rest/stimulation) indicates that thesignature of 1D structural disorder was stable, proffering the hypothesisthat differences in MD between rest and stimulation might not arise fromalterations in the one-dimensional tissue organization.

**Table 3.** Results of the 1D and 2D-3D structural disorder models fit on the MDtime-dependence.

	1D disorder		2D-3D disorder		
MD	A	D _∞_	A	t _c_ (ms)	D _∞_
Contralateral rest	0.02 ± 0.008	0.67 ± 0.004	0.08 ± 0.26	2.03 ± 6.4	0.67 ± 0.02
	AICc = -42		AIC = -21		
Contralateral stimulus	0.03 ± 0.01	0.66 ± 0.005	0.04 ± 0.33	0.29 ± 7.1	0.66 ± 0.02
	AICc = -38		AIC = -19		
Ipsilateral rest	0.02 ± 0.01	0.67 ± 0.006	0.05 ± 0.37	1.37 ± 13.1	0.67 ± 0.02
	AICc = -38		AIC = -18		
Ipsilateral stimulus	0.03 ± 0.01	0.66 ± 0.005	0.22 ± 0.36	3.37 ± 2.8	0.66 ± 0.02
	AICc = -39		AIC = -18		

As evidenced by the lower AICc values and greater precision inthe estimated coefficients, the 1D structural disorder model hasa superior fit quality for the MD time-dependent data. Theminimal differences in AICc, A, and D_∞_betweenthe rest and stimulus conditions suggest that thecharacteristics of one-dimensional structural disorder remainconsistent across both states, implying that the one-dimensionaltissue organization changes might not be responsible for the MDdrop during stimulation.

Upon analyzing MK(∆) decay (equations 3and4), the 1Dstructural disorder model demonstrated once again to be better suited. Thisconsistency in model preference across MD and MK reinforces the robustnessof the 1D structural disorder concept in characterizing the underlyingtissue microstructural features. The higher AICc values obtained fromfitting the structural disorder models to MK(∆) (Fig. 6E-H,Table 4) compared to those forMD(∆) underscore the complexity of MK time-dependence, which isinfluenced by additional factors, such as exchange. Indeed, we foundcomparable AICc values between the 1D structural disorder and theKärger exchange models (Fig. 6E, F,I,J,Table 4),reinforcing the notion that the underlying mechanisms for both models serveas confounding factors affecting the MK time-dependence. The Kärgermodel fit to MK(∆) (equation 5) yielded a shorter exchangetime during stimulation (t_ex_^stimulus^= 45.1± 12.0 ms vs. t_ex_^rest^= 53.8 ±11.1 ms contralaterally, and t_ex_^stimulus^= 51.5± 8.8 ms vs. t_ex_^rest^= 60.7 ±19.5 ms ipsilaterally), suggesting increased membrane permeability duringthe stimulus condition. However, the difference in exchange time during restand stimulation fell within the range of the estimation uncertainty (Table 4), possiblyreflecting the complex interplay between structural disorder and exchangemechanisms.

**Table 4.** Results of the 1D and 2D-3D structural disorders, and Kärgermodel kurtosis fit on the MK time-dependence.

	1D disorder		2D-3D disorder			Kärger model	
MK	A	K _∞_	A	t _c_ (ms)	K _∞_	K _0_	t _ex_ (ms)
Contralateral rest	0.33 ± 0.03	0.60 ± 0.01	0.43 ± 0.72	0.27 ± 1.36	0.66 ± 0.05	0.86 ± 0.02	53.8 ± 11.1
	AICc = -30		AICc = -11			AICc = -21	
Contralateral stimulus	0.38 ± 0.05	0.56 ± 0.02	0.17 ± 1.11	8.8e-4 ± 0.05	0.64 ± 0.07	0.85 ± 0.03	45.1 ± 12.0
	AICc = -24		AICc = -7			AICc = -17	
Ipsilateral rest	0.31 ± 0.05	0.62 ± 0.02	0.14 ±1.2	6.3e-4 ± 0.05	0.69 ± 0.07	0.86 ± 0.03	60.7 ± 19.5
	AICc = -24		AICc = -6			AICc = -18	
Ipsilateral stimulus	0.34 ± 0.03	0.61 ± 0.02	1.03 ± 0.91	1.52 ± 1.73	0.63 ± 0.06	0.87 ± 0.02	51.5 ± 8.8
	AICc = -28		AICc = -9			AICc = -23	

Consistent with the findings for MD time-dependence, the 1Dstructural disorder model exhibited lower AICc values andgreater precision in the estimated coefficients compared to the2D-3D structural disorder fit. Both structural disorder andexchange mechanisms influence MK time-dependence, which isevident in the comparable predictive abilities of the 1Dstructural disorder and Kärger models when applied to theMK(∆) data. Despite the intricate interplay betweenstructural disorder and exchange, a slight decrease in exchangetime was observed during stimulation.

## 4 Discussion

In this paper, we report brain region-specific changes in MD and MK related to a taskof forepaw electrical stimulation. In particular, we present the first evidence of adecrease in MK, paired with previously reported decreased MD (Darquié et al., 2001;Yacoub et al., 2008), associatedwith neuronal activity within the primary somatosensory cortex in the rodent brain,and an increase in MD and MK in the secondary somatosensory and subcortical areas.These modifications did not parallel changes in the BOLD signal in a systematic way,pointing to a disconnection of underlying functional contrast sources betweendiffusion weighting and BOLD.

### 4.1 Somatosensory cortical and subcortical pathways

While the primary somatosensory cortex is generally the main focus in functionalstudies of forepaw or hindpaw electrostimulation (Adamczak et al., 2010;Baltes et al., 2011;Bock et al., 1998;Brinker et al., 1999;Goloshevsky et al., 2011;Masamoto et al., 2006;Pelled et al., 2009;Spenger et al., 2000;Kim et al., 2010), adetectable BOLD response was previously reported (Bosshard et al., 2010;Boussida et al., 2017;Cho et al., 2007;Jung et al., 2019,^2021^;Keilholz et al., 2004;Weber et al., 2006;Zhao et al., 2008) in the sensorimotor and subcortical areas foundto present task-induced MD and MK changes in the current study. In addition toactivation in the contralateral S1FL, a weak BOLD response was also formerlydetected ipsilaterally (Boussida etal., 2017;Jung et al.,2019) and was attributed to somatosensory activity viainter-hemispheric sensory pathways (Jung et al., 2019). Furthermore, a positive BOLD response in thecontralateral M1 was found in several other studies (Bosshard et al., 2010;Boussida et al., 2017;Cho et al., 2007;Jung et al., 2019,^2021^;Weber et al., 2006), althoughit was construed as an overflow in some cases (Weber et al., 2006) or an antidromic stimulation ofefferent motor fibers in others (Bosshard et al., 2010;Cho et al., 2007). Nevertheless, studies using retrograde tracingtechniques have clearly demonstrated the existence of cortical projections toM1, mainly originating from the primary somatosensory region in the samehemisphere (Colechio &Alloway, 2009;Yamawakiet al., 2021). Moreover, the primary somatosensory cortex is part ofa complex intra-cortical and cortico-thalamo-cortical network which facilitatesthe flow of sensory information (Jabaudon, 2017). Another retrograde tracer injection study revealedthe existence of thalamocortical projections ascending through the caudateputamen or directly from the ventral posterolateral thalamic nucleus to theprimary and secondary somatosensory cortices, while the latter cortices wereshown to be reciprocally connected (Liao & Yen, 2008). Cortical afferents from S1 and M1 to theCPu were suggested as mediators for sensorimotor integration in the basalganglia using tracer anterograde labeling (Ramanathan et al., 2002), while somatosensoryprocessing associated with a task of whisker pad stimulation was shown totrigger hippocampal responses in electrophysiological recordings (Bellistri et al., 2013). Fromthe perspective of BOLD, positive responses were measured in S2 (Bosshard et al., 2010;Boussida et al., 2017;Jung et al., 2019,^2021^;Keilholz et al., 2004;Weber et al., 2006;Zhao et al., 2008) and Thacontralaterally (Jung et al.,2019,^2021^;Zhao et al., 2008) orbilaterally (Bosshard et al.,2010;Boussida et al.,2017;Keilholz et al.,2004), whereas a negative BOLD response was reported bilaterally inthe CPu (Boussida et al.,2017;Zhao et al.,2008). However, none of these studies reported a BOLD response in thehippocampal subfields. Globally, the entirety of this body of literaturestrongly indicates that brain activity during somatosensory processing extendswell beyond S1FL and is relevantly captured by MD and/or MK changes in ourstudy.

### 4.2 Microstructural dynamics underlying MD and MK changes in S1FL and M1

The observed MD and MK changes we report reflect the collective impact ofnumerous microscopic processes (e.g., geometric changes in the intra- andextracellular spaces resulting from cellular swelling, increased tortuosity andrestrictions in the extracellular space, decreased restrictions in theintracellular space, decreased transmembrane permeability, etc.) occurringsimultaneously across multiple spatial scales.

The measured MD drop in S1FL and M1 during the stimulus condition was previouslyreported in human (Darquiéet al., 2001;Le Bihanet al., 2006) and rodent (Abe, Tsurugizawa, et al., 2017;Tsurugizawa et al., 2013) studies, and was attributedto cellular swelling and increased tortuosity in the extracellular space. Whilethe precise mechanisms underlying an MD reduction during neural activity remainunclear, functional-induced alterations in water diffusivity may exhibit varyingpatterns contingent upon the cellular structure(s) or the spatial scale underinvestigation. For example, in the*Aplysia Californica*buccalganglia, water diffusivity was reported to increase inside neuronal soma anddecrease at the level of the whole tissue upon cellular swelling (Abe, Van Nguyen, et al., 2017;Jelescu et al., 2014),while a closer look at water diffusivity in the synaptic microenvironmentrevealed a tenfold increase near the excitatory synapses, in contrast to regionsfarther away from the synaptic site (Paviolo et al., 2022). In our study, time-dependenceanalyses of MD(∆) were expected to bring more insight into theintracellular vs. extracellular contributions behind the stimulus-induced MDchanges, but the power-law fit for structural disorder did not allow us tounambiguously disentangle between these possible contributions. A comparison ofAICc values between the structural disorder models for each condition (rest vs.stimulation) indicates that the 1D structural model provides a better fit forthe MD(∆) data in both rest and stimulation states (Table 3). In other words, theintracellular restrictions arising from variations along the main axis of axonsor cell processes such as caliber modifications and beadings seem to explainbetter the MD time-dependence in our data than the extracellular hindrancesgenerated by complex geometrical features such as undulations, branching, orinteractions between various cellular components in the 3D space. However, thesimilar microstructural coefficients (A and D_∞_) obtained fromfitting the 1D model to the MD time-dependent data at rest and duringstimulation suggest that changes in structural disorder within the intracellularcompartment may not be the main factor driving the observed MD(∆)decrease during stimulation. Conversely, the 2D-3D structural disorder alsoprovides comparable AICc values between the rest and the stimulus conditions(Table 3), thoughcertain estimated microstructural coefficients (t_c_and A) differ byup to an order of magnitude between rest and stimulation. Nonetheless, the largeuncertainties associated with these parameter estimations limit our ability todraw definitive conclusions or make further inferences about the precise natureof structural modification associated with MD/MK differences between rest andstimulation. Both types of disorder may simultaneously influence the observedMD(∆) trends, but the precise extent of their contribution remainsundetermined.

As for MK, our initial hypothesis posited that it could capture the alterationsin trans-membrane permeability linked to the regulation of imbalances induced byphysiological and biochemical processes accompanying action potentialpropagation and synaptic transmission (Bai et al., 2018,^2019^;Bellot-Saez et al., 2017;Didier et al., 2018;Kitaura et al., 2009). Notably, the genesis ofadvanced diffusion biophysical models for gray matter tissues such as NeuriteExchange Imaging (NEXI) (Jelescu etal., 2022;Olesen etal., 2022;Uhl et al.,2024) was prompted by the imperative to address the impact ofexchange between the intra- and extracellular compartments on diffusion-weightedsignals. Consequently, the imprint of exchange in gray matter is ingrainedwithin the dMRI signal. As such, the measured MK reduction in contralateral S1FLsuggested an increase in trans-membrane permeability resulting from reducedwater compartmentalization, further confirmed by the slight decrease in theestimated exchange time from MK(∆) time-dependent analyses. However, MDtime-dependence in our data is a strong indicator of the non-gaussian diffusionof water molecules generated by structural irregularities (Novikov et al., 2014).Comparisons between the fit results of structural disorder models to theMD(∆) data (Table 3)show that the 1D model provides a superior fit, along with more precisemicrostructural estimates. This signature is necessarily mirrored in the MKtime-dependence. Thus, the interpretation of MK(∆) is complicated by thesimultaneous contribution of both 1D structural disorder and exchangemechanisms, which require more advanced models and extensive datasets toseparate the exchange of these effects (Novikov et al., 2023).

### 4.3 Microstructural dynamics underlying MD and MK changes in S2 and subcortical areas

Interestingly, findings in S1FL and M1 were contrasted by the increase in MD andMK in S2 and the subcortical ROIs, possibly reflecting an opposite signature interms of water mobility across the microstructure, for example, a differentexcitatory/inhibitory balance, for the latter brain regions. Indeed,sensorimotor signals were shown to initiate both excitation and inhibitionprocesses in the various hippocampal subfields (Bellistri et al., 2013), an inhibitory postsynapticpotential was reported in various thalamic nuclei (O’Reilly et al., 2021), while the striatum isone of the main hubs for inhibitory interneurons (Fahn et al., 2011;Ramanathan et al., 2002). S2 may not be directlyassociated with an inhibitory response, but projections from both thalamicnuclei and S1 to S2 might result in both inhibitory and excitatory responses. Asfor the underlying mechanisms explaining a dominant MD increase duringinhibitory/excitatory balance, cellular shrinkage associated with neuronalhyperpolarization (Fraser &Huang, 2004) is one possible hypothesis, while reduced trans-membranewater transport in the hyperpolarized state could be at the origin of anincrease in MK. However, as suggested by a recent study focusing on the rat andhuman striatum (Cerri et al.,2024), the polarity of the functional response as measured by BOLDcannot be solely explained by neuronal activity subcortically, that is, positiveBOLD = excitatory activity and negative BOLD = inhibitoryactivity, but rather by complex neurochemical feed-forward mechanisms. Adifferent neurochemical environment across the various brain regions involved insomatosensory processing and integration could also have an impact on waterdiffusivity, and thus lead to the observed MD and MK trends. An alternativehypothesis would involve a region-dependent shift in the dominant mechanisms orwater compartmentalization influencing MD and MK. Notably, astrocytes behave asexcellent osmometers (Hellas& Andrew, 2021) and play an important role in brainexcitability (Schwartzkroin et al.,1998). The astrocytic density is higher in subcortical regions thanin the cerebral cortex in the adult rat brain (Savchenko et al., 2000) and changes in astrocytevolume were shown to be dependent on the degree of hypoosmolality of theirsurrounding medium (Lafrenaye& Simard, 2019), with a direct link between astrocyte volumeregulation and membrane permeability (Solenov et al., 2004). Nonetheless, additionalstudies are warranted to elucidate the precise mechanism at play and theirspecific relationship with MD and MK.

### 4.4 Dissociation between BOLD and diffusion changes and their respective sensitivities

As previously stated, MD and MK changes during stimulation did not parallel BOLDsignal modifications in a systematic way. In contralateral S1FL and M1, theaverage BOLD signal increase was paired by a decrease in MK and/or MD, whileipsilateral S1FL presented a decrease in MD in the absence of detectable BOLDsignal variations. On the other hand, a weak negative BOLD signal wasaccompanied by strongly significant elevations of MD and MK in the CPu. In theremaining brain regions, the trends were dissociated: the BOLD signal and MDincreased in contralateral S2, while no significant BOLD changes were found inipsilateral S2, ipsilateral CPu, and bilateral Tha or Hip in the presence of anincrease in MD and/or MK. The absolute amplitude of the BOLD signal changesscaled with the expected functional involvement of each area, with the highestresponse in S1FL (1.2%) and gradually less in S2 (0.3%), M1 (0.2%), and CPu(0.1%), although whether the order between S2, M1, and CPu reflects the actualstrength of functional activity is not known. Intriguingly, the absoluteamplitude of the significant MD and MK changes was overall above 1%, except forMD in the ipsilateral S1FL. The absolute maximum amplitude changes for MD wereweaker in S1FL and M1 (1.1%/1.2%) than in areas of MD increase, such ascontralateral S2 or Tha (1.5%/1.9%). On the other hand, contralateral S1FLshowed a marked absolute MK variation (maximum of 4.9%) while the absoluteamplitude of the significant changes in Tha and Hip was reduced by half (maximumof 2.4%). While contralateral S1FL is undoubtedly the area of strongestexcitatory activity in this task, it remains to be established if the comparableMD amplitude changes between S1FL and the subcortical regions are the result ofcompeting mechanisms that contribute to either an increase or decrease in MD, ora possibly strong inhibitory response in subcortical regions.

Measuring significant diffusion changes below the BOLD detection thresholdindicates a higher sensitivity for MD and MK to brain activity, although it ispossible that the neurovascular responses in the subcortical areas were too weakto be detected with our experimental setup. Indeed, the acquired dMRI andBOLD-fMRI images reflect the sensitivity profile of a surface coil, with reducedSNR in the deeper brain regions as compared to the cortex. However, both GE-EPIand PGSE-EPI were affected by this profile, with the spin-echo diffusion imagespossibly even more affected due to larger flip angles and relative inefficiencyof the refocusing pulse far from the coil. Furthermore, the GE-EPI sequence wasoptimized for BOLD sensitivity, following previously established and recommendedprotocols (Grandjean et al.,2023;Reynaud et al.,2019). Beyond GLM voxel-wise analyses, we attempted to match thesensitivity of BOLD and MD/MK by averaging the BOLD signal across all rest andstimulus intervals for each anatomical ROI. In spite of this, we report anincrease in MD and MK in the thalamus and the hippocampus, in the absence of anotable BOLD signal change.

Furthermore, changes in the diffusion metrics remained specific to regionsinvolved in the somatosensory processing and integration, as no MD or MK changeswere observed in cortical regions such as ACC, RSC, PPC, or M2 which were alsodevoid of a BOLD response.

### 4.5 Limitations—dMRI

The fast kurtosis approach (Hansen,Lund, et al., 2016) used to estimate MD and MK in a scan timecompatible with a functional block design is certainly associated with higherestimation uncertainty as compared to the full kurtosis tensor estimation. Inpractice, a ‘classical’ protocol for diffusion and kurtosis tensorestimation comprises approximately 30 directions per b-value, well superior tothe nine directions per b-value acquired in this study. In addition, the higherintrinsic sensitivity to noise for the kurtosis metrics led to a relativelylarge MK uncertainty, which also translated into fewer ROIs displaying asignificant change in MK as compared to MD. Thus, the analysis of time-dependentMK(∆) was likely confounded not just by concomitant contributions betweenstructural disorder and exchange, but also by the limited precision of fast MKestimates. Ideally, future studies would employ microstructural biophysicalmodels, such as NEXI (Jelescu etal., 2022;Olesen etal., 2022;Uhl et al.,2024), or more complex models simultaneously accounting for bothstructural disorder and exchange mechanisms (Novikov et al., 2023), to enable access tocompartment-specific water diffusivity changes associated with neural activity.However, the use of multiple dMRI measurements to estimate the parameters ofinterest limits this approach to task fMRI with long block designs (56 s perepoch in our case, 3 epochs per run) producing a sustained activity over asufficiently long duration. Such limitations restrict our ability to resolverapid microstructural changes associated with the neural response anddistinguish between the various physiological mechanisms underlying thetransient water diffusivity changes. Consequently, the observed MD and MKdifferences between the rest and stimulation states in this study likely reflectmore sustained or cumulative microstructural changes. Faster dMRI acquisitionschemes, as for example based on isotropic encoding (Nunes et al., 2019,^2021^;Spencer et al., 2024), enablehigher temporal resolution acquisitions and could be used in future studies,although their unclear definition of the diffusion time poses additionalchallenges for the time-dependence analyses.

The low number of measurements per functional run for MD and MK estimation alsolimited the statistical power of our data, as compared to BOLD where manysamples are acquired with high temporal resolution. Therefore, the voxel-wiseGLM analysis typical for BOLD-fMRI did not yield any significant results for MDor MK. As a result, we compensated for the low number of available measurementsby averaging over anatomical ROIs. The voxel-wise b = 0 SNR ranged from10 (thalamus) to 20 (S1FL), which translates into good accuracy and precision ofMD and MK estimates (see Fig. 2 inHansen, Lund, et al., 2016) in most ROIs, except for the thalamus.However, the ROI averaging over 100–1400 voxels ensured the precision waswell improved in all cases. The same ROI averaging for the BOLD signal wasperformed to make the statistical results from the two contrasts more directlycomparable.

Another limitation is the possible BOLD-like contribution to MD and MK estimates,that is, driven by changes in blood volume and oxygenation that causesusceptibility changes in the surrounding tissues. While working with MD and MK,rather than raw diffusion-weighted signals, substantially reducesT_2_-weighting effects that could reflect BOLD contrast, it isimportant to note that our experimental design remains somewhat susceptible tothese influences. As illustrated inFigure S3, the BOLD response function is notconstant during the stimulation time-window and extends into the rest intervalfor approximately 4 s post-stimulus. The dynamic nature of the BOLD responseresults in a variable T_2_-weighting bias across the dMRI measurements.Methods such as the Incomplete Initial Nutation Diffusion Imaging offer apromising approach to separate T_2_effects from diffusion measurements(Ianuş & Shemesh,2018;Nunes et al.,2018) and could eliminate potential biases in the quantification ofMD and MK that arise from time-varying T_2_-weighting. Nevertheless,the use of an ultra-high magnetic field strength (here, 14.1T) results insubstantially shorter T_2_relaxation times for blood, thussignificantly diminishing the direct contribution of intravascular water to themeasured diffusion signal. Conversely, the use of higher magnetic fieldstrengths introduces a new set of challenges. In particular,susceptibility-induced background field gradients around blood vessels becomemuch more significant and their interaction with the diffusion-weightinggradients can lead to an increase in apparent diffusivity (Pampel et al., 2010). We note,however, that in the case of positive BOLD, we measured a decrease in MD ratherthan an increase in MD, and we also report MD and MK changes in areas where ameasurable BOLD response was absent. The proof that task-driven MD and MKchanges are not dependent on vascular response but are instead associated withchanges in cellular microstructure remains valid.

Finally, we emphasize that reporting the largest amplitude change in MD and MKacross the various diffusion times allowed us to identify the optimum diffusiontime value for maximum sensitivity to region-specific functional-inducedchanges. Overall, the optimal diffusion time should be tuned to thecharacteristic exchange time of the system. Consequently, it was not surprisingthat we observed the majority of maximum amplitude changes in MD and MK withinthe range of 15–25 ms (Jelescu et al., 2022). For whole-brain studies at a single diffusiontime, a value of around 15 ms represents the best compromise in terms ofsensitivity to functional MD and MK changes across brain regions. However,various types of stimuli, differences in the tissue type investigated (WhiteMatter vs. Gray Matter), or the presence of a pathological state might promptthe need for a different acquisition protocol to enhance the functionalsensitivity of MD/MK.

### 4.6 Limitations–BOLD-fMRI

Factors such as magnetic field strength (Gati et al., 1997), echo time (Grüne et al., 1999),and temporal SNR (tSNR) (Murphy etal., 2007) all influence BOLD sensitivity. In this study, weleveraged the advantages of an ultra-high magnetic field system to enhance BOLDsensitivity, and the echo time was optimally set according to a recentlypublished consensus protocol for functional connectivity analysis in the ratbrain (Grandjean et al.,2023). Nonetheless, lower T_2_* in subcortical brainregions (Boussida et al.,2017), resulting from differences in vascular organization (Sloan et al., 2010) andsusceptibility effects as compared to cortical areas, might prompt the need fora different echo time in order to maximize BOLD sensitivity subcortically. Theuse of a surface transceiver RF coil in our study led to a notable tSNRreduction in the deeper structures of the brain, possibly obscuring weaker BOLDchanges. However, the same penalty applied for the diffusion-weighted images.New approaches for the analysis of BOLD-fMRI time-series employing responsefunctions tailored (Gonzalez-Castillo et al., 2012;Schilling et al., 2018) by region, task, and/orsubject could be used in the future for a more robust detection of voxel-wisesignificant BOLD signal changes beyond the main activated brain regions.

## 5 Conclusions

To conclude, in this study we have shown an MD and MK reduction during brain activityin contralateral S1FL consistent with changes in cellular morphology and watertransport mechanisms, paired with a positive BOLD response. As the MD and MK changesbetween the rest and stimulus conditions did not parallel the BOLD signal dynamicsin a systematic way in all the investigated brain regions, the current studydemonstrates the potential of MD and MK to provide complementary functionalinformation to BOLD across multiple cortical and subcortical areas in the rat brain.Remarkably, MD and MK detected functional-induced changes in areas reported in theliterature as part of the somatosensory processing and integration pathways, withoverall higher sensitivity than BOLD. Future research endeavors will focus on adeeper investigation of the underlying mechanisms behind the elevation in MD and MKassociated with neural activity, particularly as observed in S2 and subcortically.Calcium imaging, along with chemogenetic and optogenetic neuromodulation techniques,enables precise targeting of specific cell types in vivo, facilitating theassessment of contributions from various populations, including glial cells andexcitatory and inhibitory neurons (Kim& Jun, 2013;Markicevic et al., 2021;Sancataldo et al., 2019). When combined with advanced super-resolutiontechniques that visualize the organization of the extracellular space at thecellular level (Godin et al.,2017;Gwosch et al.,2020;Tønnesen etal., 2018), these methods can provide a more comprehensive understandingof the brain’s microstructure-function relationship during neural activity,particularly in relation to the dMRI measurements.

## Supplementary Material

Supplementary Material

## Data Availability

The raw data generated for this study are available in the following repository:https://doi.org/10.5281/zenodo.14793797.
